# Calculated globulin can be used as a screening test for antibody deficiency in children and adolescents

**DOI:** 10.3389/fimmu.2024.1495564

**Published:** 2024-10-23

**Authors:** Cristina Frias Sartorelli de Toledo Piza, Carolina Sanchez Aranda, Dirceu Solé, Stephen Jolles, Antonio Condino-Neto

**Affiliations:** ^1^ Division of Allergy, Immunology and Rheumatology, Department of Pediatrics, Federal University of São Paulo, São Paulo, Brazil; ^2^ Immunodeficiency Centre for Wales, University Hospital of Wales, Cardiff, United Kingdom; ^3^ Department of Immunology, Institute of Biomedical Sciences, University of São Paulo, São Paulo, Brazil

**Keywords:** antibody deficiency, hypogammaglobulinemia, primary immunodeficiency, secondary immunodeficiency, calculated globulin (CG)

## Abstract

**Purpose:**

Calculated globulin (CG, total protein minus albumin levels) correlate well with IgG levels and has been proposed as a suitable screening method for individuals with primary antibody deficiencies (PADs). We aimed to show the correlation of CG with IgG levels in children and adolescents, utilizing a common method for albumin measurement, bromocresol green.

**Methods:**

Individuals from two Allergy and Immunology clinics were invited to participate. Inclusion criteria were age < 18, stable conditions, and signed informed consent. We included 1084 individuals. Immunoglobulin G values were determined by immunoturbidimetry; the colorimetric bromocresol green method and the Architect Biuret method were utilized for the albumin and total protein (TP) measurements, respectively.

**Results:**

A total of 1084 individuals were included in the analysis and divided into 4 age groups (0 to <1 year, 1 to <4 years, 4 to <10 years, and 10 to <18 years). For all patients, the mean age was 6.1 (± 5) years old, the mean IgG was 9.4 (± 4.7) g/L, and CG was 23.7 (± 5.9) g/L. The most frequent diagnosis were respiratory allergies, followed by inborn errors of immunity. IgG and CG varied according to age group. Cutoff values for hypogammaglobulinemia varied from 13.8 g/L in children < 1 year to 23.1 g/L in children and adolescents aged 10 to <18 years. CG sensitivity varied from 70.9% in children aged 1 to <4 years old to 95.8% in children 4 to <10. Specificity ranged from 87.5% in children 4 to <10 years old to 100% in children and adolescents aged 10 to <18 years.

**Conclusion:**

CG is a suitable screening test for hypogammaglobulinemia in children less than 18 years of age.

## Introduction

Inborn errors of immunity (IEIs) encompass a diverse array of disorders, with over 485 unique genetic conditions and related health issues identified to date (1). Discoveries of the genetic defects underlying IEIs are occurring at an unprecedented pace. As a result, the clinical phenotypes associated with these conditions are becoming more precisely defined. This growing clarity highlights the significant health burden these diseases pose collectively. The prevalence of IEIs is currently estimated to be between 1 in 1,000 and 1 in 5,000 individuals (2).

Despite significant progress in research, including genetic sequencing and molecular diagnosis, that have enhanced our understanding of the immune system and improved the quality of life for individuals with primary immunodeficiencies (PIs), awareness of PIs remains a crucial concern for both physicians and the general public (3).

Accurate prevalence estimates of IEIs are affected by underdiagnosis, underreporting, and potential mortality before diagnosis, particularly in certain infant cases. Underdiagnosis may occur due to limited awareness, insufficient newborn screening, absence of family history or carrier testing, and asymptomatic IEIs. Patients encounter significant issues such as diagnostic delays and misdiagnosis, both of which delay appropriate treatment (4).

Primary antibody deficiency (PAD), the most frequently occurring type of IEI, is characterized by a failure to produce clinically significant levels of immunoglobulin, predominantly IgG (5). The symptoms, severity, and typical onset of PADs differ, and in addition to a heightened frequency of infections, they may also lead to complications associated with autoimmunity and malignancy (5). PADs are amenable to IgG replacement therapy (IgRT), which may prevent later organ dysfunction, reduce morbidity and mortality, and improve quality of life (6, 7).

Calculated globulin (CG, total protein minus serum albumin content) has been proposed as a reliable screening marker for early PAD diagnosis in adults (8, 9). The addition of automated calculation of CG when running total protein and albumin measurements for other conditions has been suggested (10). We have previously reported the correlation of CG, obtained from protein electrophoresis (PE) measurements, with IgG levels in children, adolescents (11), and adults (10). A significant number of patients with primary antibody deficiencies (PAD) produce seemingly adequate levels of IgG but fail to generate a protective response to pathogens, as seen in specific antibody deficiency (SAD). This group of patients may go undetected by CG screening and cannot be identified through direct IgG measurement alone. Physicians should be aware of this and consider additional testing when suspecting this condition.

There are several methods for the determination of albumin used in clinical practice. Dye-binding methods, such as bromocresol green, are the most commonly used, due to their precision and speed, compared to other methods such as PE (12).

This study aimed to assess the correlation between calculated globulin levels and serum IgG levels in children under 18 years old, using the bromocresol green method for albumin measurement. We demonstrate how this approach can aid in detecting hypogammaglobulinemia in children and lay the groundwork for future automated screening in large routine diagnostic laboratories, which is currently being initiated in Brazil.

## Methods

### Participant inclusion procedures

The University of São Paulo and the Federal University of São Paulo Ethics Committees approved the protocol (approval numbers 3.340.392 and 3.499.511, respectively) according to the rules and regulations of the Brazilian Ministry of Health and the Declaration of Helsinki. Patients attending two different Allergy/Immunology centers in São Paulo state, Brazil, were invited to participate, regardless of consultation purpose. The inclusion criteria included outpatients under 18 years with clinically stable conditions whose parents provided informed consent. The exclusion criteria were age above 18, unstable clinical conditions, and lack of informed consent.

Written informed consent was obtained before the inclusion of participants and blood collection.

A 5 mL blood sample was collected from each individual for laboratory analyses. Patients were free to choose the laboratory in either center. All laboratories were contacted to ascertain the measurement methods. The laboratories were accredited according to the Associação Brasileira de Normas Técnicas (ABNT NBR ISO 15189) (13) and the Brazilian Society of Clinical Pathology (PALC) (14).

### Laboratory measurements

Immunoglobulin G values were determined by immunoturbidimetry (Roche COBAS 6000, Roche Diagnostics International Ltd., CH-6343, Rotkreuz, Switzerland).

The colorimetric bromocresol green (BCG) method and the Architect Biuret method (16200 Abbott Analyzer; Abbott Diagnostics) were utilized for the albumin and total protein (TP) measurements, respectively. CG values were obtained by subtracting albumin levels from the total protein values.

IgG reference values were based on Adeli et al. (15).

### Statistical analysis

The assumptions of normality of the data distribution and homogeneity of variance were checked using the Shapiro–Wilk and Levene’s tests, respectively. One-way ANOVA followed by Bonferroni *post hoc* correction was used to compare the IgG and CG levels between the different age groups. Simple linear regression models determined the explained variance in IgG levels based on CG levels (16, 17).

Next, receiver operating characteristic curves were constructed to identify the CG cutoff values (18) to discriminate between patients with below-reference and normal IgG levels. Individuals with IgG values below 1.5 g/L (< 1 year), 3.2 g/L (1 to < 4 years), 5.0 g/L (4 to < 10 years), and 6.0 g/L (10 to < 18 years) were classified as below reference. The accuracy of the CG cutoff values to discriminate between patients with below-reference and normal IgG levels was verified by sensitivity (true positive rate - correct identification of patients with below-reference IgG levels) and specificity (true negative rate - correct identification of patients with normal IgG levels) tests; construction of receiver operating characteristic curves; and analysis of the areas under the curves (AUCs) and their respective 95% confidence intervals. The accuracy of the discriminant value was interpreted based on the AUC and classified as perfect (AUC = 1), exceptional (0.9 ≤ AUC < 1), excellent (0.8 ≤ AUC < 0.9), acceptable (0.7 ≤ AUC < 0.8) or poor (AUC < 0.7), considering that the AUC is not significantly different from that obtained at random for AUC values ≤ 0.5. To confirm the discriminant score, the Youden index was calculated as the highest value observed for the following operation: sensitivity + specificity – 1.

All the analyses were carried out using PASW statistics 26.0 software (SPSS Inc., Chicago, USA), with a significance level (α) of 5% (P < 0.05).

## Results

A total of 1084 individuals participated in this study. IgG and CG levels increased with age, with significant differences among age groups (p < 0.001 for all comparisons) (**Table 1**).

**Table 1:** Immunoglobulin G and CG levels according to age groups.

Age group	N	Mean age (years) (± SD)	Mean IgG (g/L) (± SD)	Mean CG (g/dL) (± SD)	Male sex
<1 year	147	0,49 (± 0,27)	0,27 (± 4,35)	16,79 (± 4,25)	53,7%
1 year to < 4 years	303	1,87 (± 0,82)	0,82 (± 8,36)	22,68 (± 4,24)*	50,2%
4 years to < 10 years	325	6,13 (± 1,76)	1,76 (± 10,49)	25,26 (± 4,19)*	49,8%
10 years to < 18 years	309	12,90 (± 2,30)	2,30 (± 11,82)	26,38 (± 6,53)*	46,0%
All ages	1084	6,13 (± 5,02)	5,02 (± 9,08)	23,71 (± 5,86)	49,5%

*p<0.001 when compared to last group.

**Figure 1:**
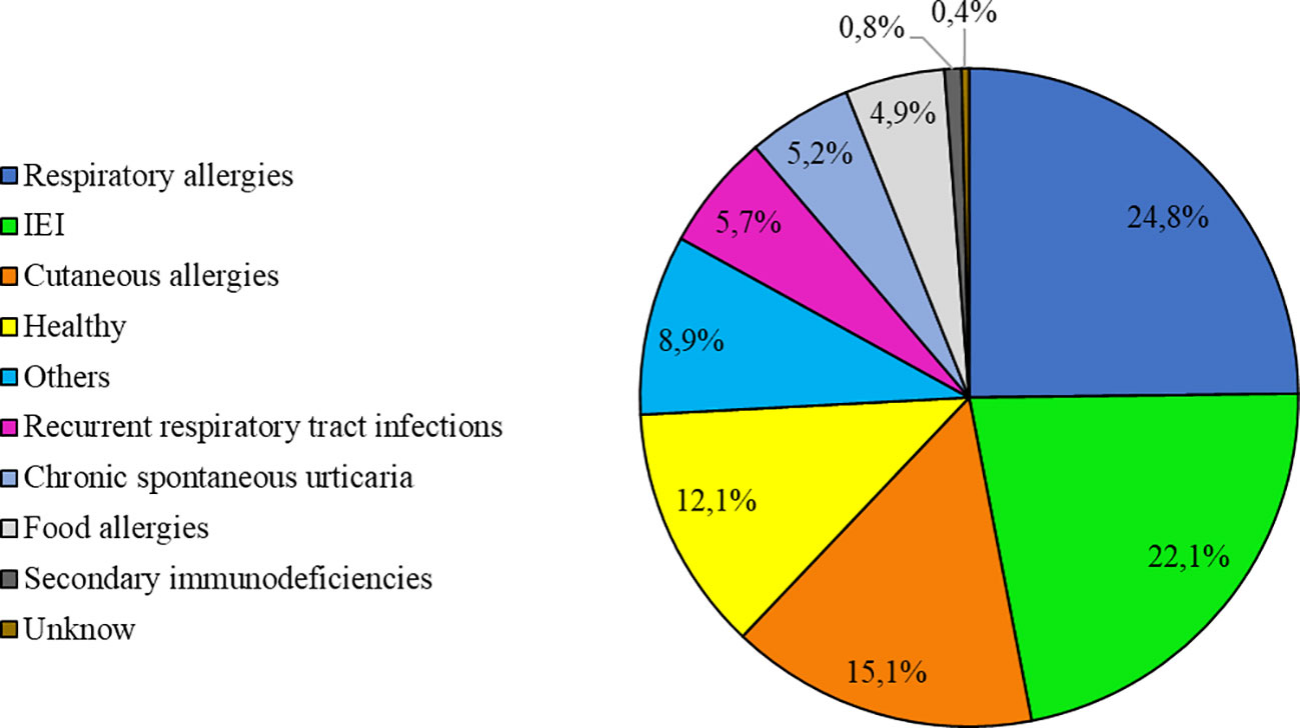
Most common diagnoses (n = 1084).

The most common diagnoses were respiratory allergies (24.8%) and primary immunodeficiencies (22.1%) (see **Figure 1** for other diagnoses).

Of the 249 patients with an IEI, 115 patients were receiving IgG replacement therapy (IgRT). Forty seven patients had a diagnosis of hypogammaglobulinemia, but were not receiving IgRT at the time of sample collection.

**Figure 2:**
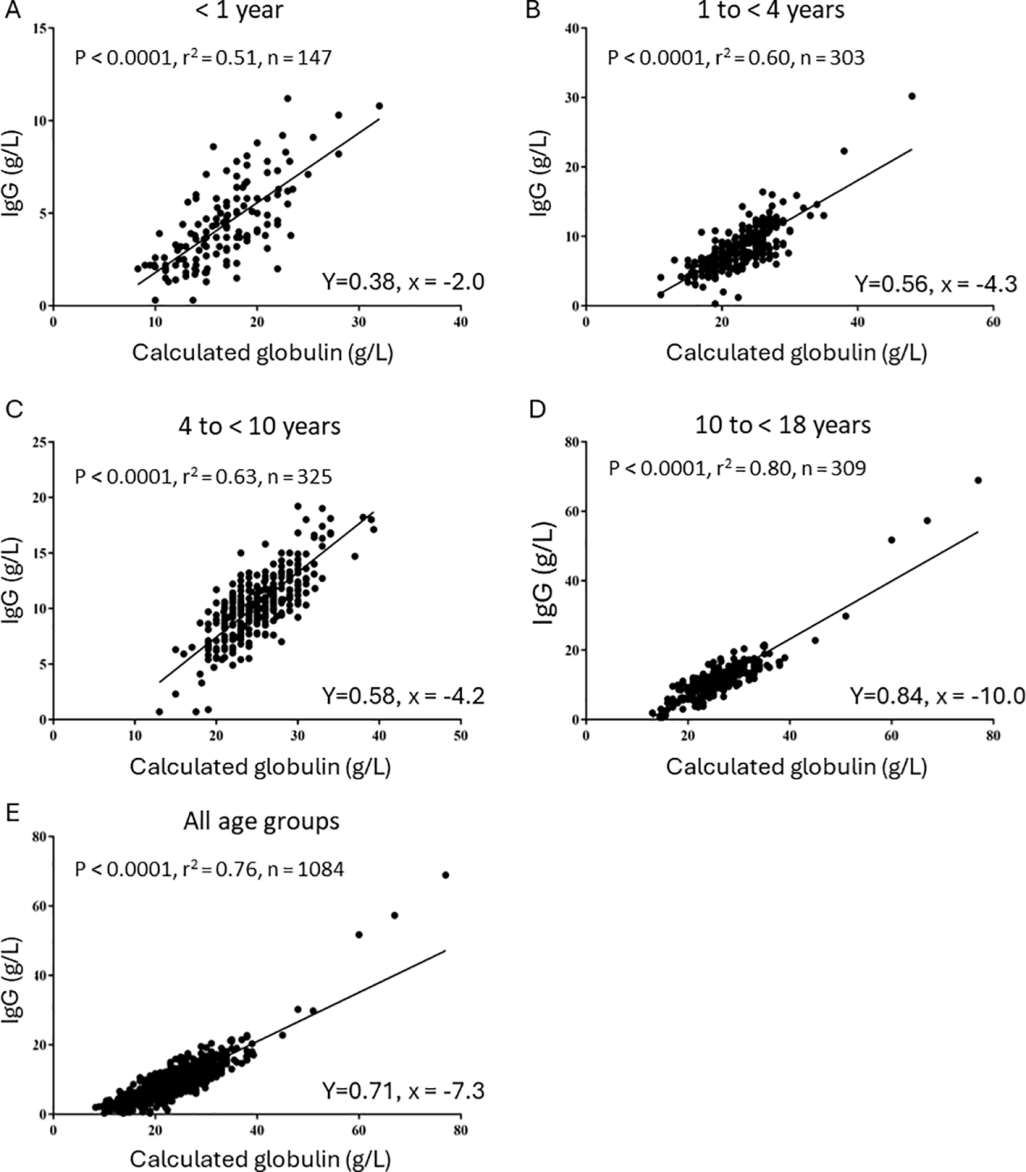
Linear regression models for the correlation between CG and IgG levels. **(A)** 0 to 1 year old. **(B)** 1 year to < 4 years. **(C)** 4 years to 10 years. **(D)** 10 to < 18 years. **(E)** All ages.

A positive and statistically significant association between IgG levels and CG levels was observed in all age groups, separately and in the combined analysis of all patients (P < 0.0001 for all, **Figure 2**). CG levels significantly explained part of the variance (%) of IgG levels in all age groups analyzed— < 1 year, 51% (**Figure 2A**); 1 to < 4 years, 60% (**Figure 2B**); 4 to < 10 years, 58% (**Figure 2C**); and 10 to < 18 years, 84% (**Figure 2D**)—as well as when all age groups were analyzed together (71%, **Figure 2E**).

For samples with below-reference or normal IgG levels, the predictive power of CG cutoff levels was classified as excellent to exceptional (AUC from 0.84 to 0.95, P < 0.01 for all), with the AUC being significant and having acceptable accuracy for all age groups (**Table 2**). The sensitivity and specificity ranged from 70.6% to 100%. In addition, excellent accuracy was observed for the cutoff obtained when the age groups were analyzed together (AUC = 0.86, P < 0.001, sensitivity = 77.0%, and specificity = 78.7%).

**Table 2:** AUC, 95% CI, sensitivity and especificity for CG as a screening test for hypogammaglobulinemia.

Age Group	AUC	95% CI	P value	Cut-off value (g/L)	Sensitivity	Especificity	Samples	
							Below reference*	Normal
<1 year	0.86	0.76 - 0.95	0.003	13.80	80.1%	83.3%	6	141
1 year to < 4 years	0.84	0.73 - 0.96	0.002	20.25	70.6%	85.7%	7	296
4 years to < 10 years	0.96	0.92 - 1.00	< 0,001	19.85	95.6%	87.5%	8	317
10 years to < 18 years	0.95	0.92 - 0.98	< 0,001	23.10	76.3%	100.0%	26	283
All ages	0.86	0.82 - 0.89	< 0.001	20.25	77.0%	78.7%	47	1037

*Number of samples with serum IgG values below reference for each age group.

## Discussion

Delays in the diagnosis and treatment of IEIs can lead to increased morbidity and mortality (19) and overall elevated treatment costs (20). Several published articles highlight that CG level can serve as an indicator of IgG levels in adults (8, 9, 21, 22), but few studies have evaluated this marker in children and adolescents.

In this report, we show that CG may serve as a screening method for children undergoing tests for other reasons. We demonstrate that cutoff values are variable, depending on the age group, from 13.8 g/L in children under 1 year of age to 23.1 g/L in children and adolescents from 10 to under 18 years of age. Sensitivity and specificity also varied according to age. In particular, the lowest sensitivity was 70.6% for samples from patients aged 1 to 4 years old, for unclear reasons.

Our group recently published a study correlating CG and IgG levels using PE for total protein and albumin measurement (11) in 1235 samples from children and adolescents less than 18 years old. The results showed that CG levels and cutoff values varied according to age group. The sensitivity varied from 75% in patients aged 2 to 3 years to 100% in patients aged 0 to 1 years and those aged 6 to 9 years, while the specificity varied from 62.7% in children younger than 1 year to 100% in adolescents aged 13 to < 18 years.

Using the bromocresol green methods for albumin measurement, Spiridonova et al. (23) evaluated 497 children (median age 8.3 years, interquartile range 2.7 to 15) for correlations between IgG and CG levels in different age groups. The best results for predicting low IgG levels were observed for a CG cutoff value of 19 g/L. The study revealed 100% sensitivity and 89% specificity for children between 0 and 17 months of age and 91% sensitivity and 90% specificity for children between 18 months of age and 2 years of age. It is difficult to compare these results with those showed in our study, since we analyzed different age groups. For the same reason, we could not compare the results with bromocresol green to our previous study using PE.

The above studies showed that CG can be a useful screening marker for children and adolescents. Interestingly, the cutoff values for CG varied based on the age group, reflecting the different IgG levels associated with each age range.

This is the largest report of CG in children and adolescents using the bromocresol green method for albumin measurement, the most common method used worldwide. Other methods used for albumin and total protein measurements, such as our own previous study (11) using PE, are less frequently used, and in the case of PE, more labor intense.

Notably, our research results have certain limitations. The study primarily included patients who frequented allergy/immunology clinics and often had a history of repeated infections. As a result, the accuracy of the findings may differ in other demographic groups. Certain diseases may alter albumin serum concentrations (nephrotic syndrome, hepatic insufficiency etc.), and increase or decrease α and β globulins (hyperliproteineemia, metastatic malignancy, iron deficiency anemia etc.) (24). On the other hand, in infectious or inflammatory disorders, globulins may increase significantly and elevate the CG fraction, eventually yielding false negative results. In these cases, calculated globulin accuracy may be compromised. Additionally, a significant number of patients with PAD produce seemingly adequate levels of IgG but fail to generate a protective response to pathogens, as seen in SAD. This group of patients may go undetected by CG screening and cannot be identified through direct IgG measurement alone. Physicians should be aware of this condition and consider additional testing when suspecting those conditions.

If an inborn error of immunity is suspected, either because of a calculated globulin level, clinical history or other, referral to an experienced immunologist is recommended.

In conclusion, CG is a valuable screening marker for detecting hypogammaglobulinemia in individuals younger than 18. CG should not be used for patients suspected of having hypogammaglobulinemia since Ig measurements are cheaper and yield conclusive results, but rather as an opportunistic screening for patients undergoing albumin and total protein measurements for other diagnoses. We recommend that automated CG calculations be incorporated into routine practice when performing total protein and albumin measurements. This initiative is already being implemented in major diagnostic laboratories across Brazil, with the support of the Brazilian Society of Clinical Pathology, the Brazilian Association of Asthma, Allergy, and Immunology, and the Brazilian Society of Pediatrics.

## Data Availability

The raw data supporting the conclusions of this article will be made available by the authors, without undue reservation.
